# Maternal Diet Quality Assessed Using the Korean Healthy Eating Index and Risk of Small-for-Gestational-Age Infants: Findings from the Mothers and Children’s Environmental Health (MOCEH) Study

**DOI:** 10.3390/nu17193056

**Published:** 2025-09-25

**Authors:** Won Jang, Minji Kim, Eunhee Ha, Hyesook Kim

**Affiliations:** 1 Department of Food Science and Nutrition, Dongseo University, Busan 47011, Republic of Korea; jangwon@gdsu.dongseo.ac.kr; 2 Institute for Better Living, Wonkwang University, Iksan 54538, Republic of Korea; 3 Department of Nutritional Science and Food Management, Graduate Program in System Health Science and Engineering, Ewha Womans University, Seoul 03760, Republic of Korea; 0304minji@naver.com; 4 Graduate Program in System Health Science and Engineering, College of Medicine, Ewha Womans University, Seoul 07804, Republic of Korea; eunheeha@ewha.ac.kr; 5 Department of Environmental Medicine, School of Medicine, Ewha Womans University, Seoul KS013, Republic of Korea; 6 Institute of Ewha-SCL for Environmental Health (IESEH), College of Medicine, Ewha Womans University, Seoul 07804, Republic of Korea; 7 Department of Medical Science, College of Medicine, Ewha Medical Research Institute, Ewha Womans University, Seoul 03760, Republic of Korea; 8 Department of Food and Nutrition, Wonkwang University, Iksan 54538, Republic of Korea

**Keywords:** small for gestational age (SGA), pregnant women, diet quality, Korean Healthy Eating Index (KHEI), birth cohort study

## Abstract

**Background**: Maternal diet quality during pregnancy may significantly influence fetal growth and birth weight. **Objectives**: This study aimed to assess the dietary quality of pregnant women in Korea and investigate its association with the risk of delivering a small-for-gestational-age (SGA) infant. **Methods**: A total of 1158 pregnant women and their newborns were recruited from the Mothers and Children’s Environmental Health (MOCEH) 2006–2010 cohort. Maternal dietary intake during gestational weeks 12–28 was assessed using a validated food frequency questionnaire. The nutrient adequacy ratio (NAR), mean adequacy ratio (MAR), and Korean Healthy Eating Index (KHEI) were employed to evaluate dietary quality. Birth outcomes were obtained from hospital records, and logistic regression analyses were performed to examine associations between maternal dietary quality and SGA risk. **Results**: Higher KHEI scores were significantly associated with increased nutrient intake, with the exception of fat, and demonstrated a positive association with the NAR of 15 nutrients. After adjusting for covariates, women in the highest KHEI quartile exhibited a significantly lower risk of SGA birth than those in the lowest quartile (adjusted odds ratio: 0.448; 95% confidence interval: 0.201–0.997; P-for-trend = 0.031). **Conclusions**: Enhanced maternal diet quality, as measured using the KHEI, is associated with improved nutrient intake and a reduced risk of SGA births among Korean pregnant women. These findings underscore the need for public health strategies that promote high-quality diets during pregnancy to improve birth outcomes.

## 1. Introduction

Small-for-gestational-age (SGA) infants are defined as those with a birth weight below the 10th percentile for their gestational age. Unlike the non-standardized term “low birth weight,” which encompasses both premature and growth-restricted infants, SGA serves as a more specific indicator of fetal growth restriction [1]. SGA infants experience higher perinatal mortality rates than those with normal birth weight [2,3]. Additionally, they are at an increased risk of postnatal growth delays [4,5,6] and neurodevelopmental impairments in childhood, such as learning disabilities, motor and cognitive deficits, and challenges in social–emotional and adaptive functioning [7,8,9]. In the long term, these infants also face an elevated risk of chronic diseases, including cardiovascular disease and type 2 diabetes [10]. Furthermore, the delivery of an SGA infant has been associated with an increased risk of cardiovascular disease in the mother [1].

Maternal nutrition during pregnancy is widely recognized as a critical factor influencing not only SGA infants but also various adverse birth outcomes [11]. Maternal nutritional status influences not only the intrauterine environment but also the long-term health trajectories of both the mother and child. Inadequate or excessive nutrient intake before and during pregnancy has been linked to suboptimal outcomes, including fetal growth restriction, low birth weight, and other complications that may impact maternal well-being and child development [12,13,14,15].

Although numerous studies have investigated the effects of individual nutrients or specific food items on pregnancy outcomes [16,17,18], such approaches may insufficiently capture the complexity of overall maternal diet quality [11]. Consequently, emphasis on the use of composite dietary quality indices that consider the cumulative effects of multiple dietary components has grown [19,20]. Several validated indices have been developed to quantify overall dietary patterns, including the Healthy Eating Index (HEI) [21], Mediterranean Diet Score [22], Dietary Inflammatory Index [23], and Dietary Approaches to Stop Hypertension diet score [24]. These indices have been utilized in pregnancy-related studies to explore associations with gestational weight gain, gestational diabetes, and various birth outcomes. However, evidence linking maternal dietary quality to SGA risk, particularly in non-Western populations, remains limited.

To address this gap, the Korean Healthy Eating Index (KHEI) presents a culturally relevant yet structurally comparable dietary quality index [25]. Originally developed for the Korean adult population, the KHEI integrates both international principles of healthy eating and Korea’s national dietary guidelines [25]. Therefore, it provides a valuable framework for assessing maternal dietary patterns within an East Asian context and contributes to a broader understanding of how culturally adapted indices can inform maternal nutrition research.

This study aimed to assess the dietary quality of pregnant women using a culturally relevant dietary quality index and examine its association with the risk of delivering an SGA infant.

## 2. Materials and Methods

### 2.1. Study Participants

The present study forms part of the Mothers and Children’s Environmental Health (MOCEH) initiative, a community-based prospective birth cohort study initiated in Korea in 2006. It was conducted across three urban centers: Seoul (metropolitan area), Ulsan (industrial area), and Cheonan (urban area) and approved by three institutional review boards: Ewha Womans University School of Medicine, Dankook University Hospital, and Ulsan University Hospital. Informed consent was obtained from all participants [26].

**Figure 1:**
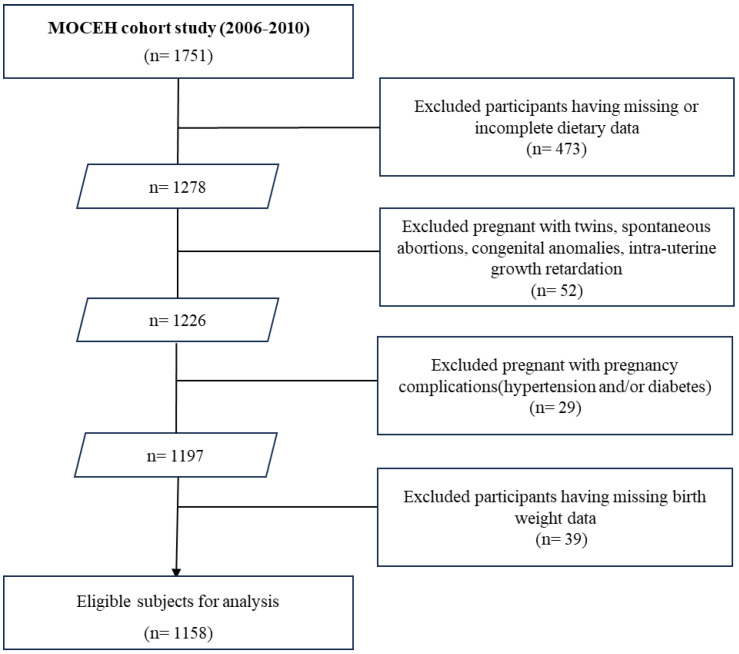
A flowchart of study population.

The study was conducted between August 2006 and December 2010, recruiting 1751 pregnant women at 12–18 weeks of gestation from three university hospitals. Of these, 473 women were excluded owing to missing or incomplete dietary intake data. Additionally, 52 participants with twins, spontaneous abortions, congenital anomalies, or intrauterine growth retardation, as well as 29 individuals with pregnancy complications (hypertension and/or diabetes), were also excluded. Thereafter, 39 participants with missing birth weights were excluded from the remaining 1197 individuals, resulting in a final cohort of 1158 participants (Figure 1).

Trained personnel collected demographic information from participants, including age (years), pre-pregnancy weight (kg), height (m), education level (high school or lower, lower than university, or university or higher), household income (<2,000,000 USD, 2,000,000–4,000,000 USD, or ≥4,000,000 USD), parity, and health-related behaviors (e.g., nutritional supplement intake [yes/no]), using structured questionnaires. Pre-pregnancy body mass index (BMI) was calculated by dividing weight by the square of self-reported height. Data on newborn characteristics, including sex, gestational age (days), birth length (cm), and birth weight (g), were extracted from medical records. Maternal gestational age was estimated based on the last menstrual period and confirmed via ultrasound examination. Cotinine levels were measured using the high-performance liquid chromatography–isotope dilution tandem mass spectrometry method (Agilent 6410 Triple Quad LCMS, Agilent Technologies, Santa Clara, CA, USA), with data adjusted for urinary creatinine concentration to account for urinary volume [27]. SGA infants were defined as those with birth weights below the 10th percentile for gestational age, based on sex-specific growth curves for Korean singleton fetuses. Gestational age refers to the duration (completed weeks) from the first day of the mother’s last menstrual period to the day of delivery [28].

### 2.2. Dietary Assessment

Nutritional and dietary information of pregnant women was collected using a semiquantitative self-administered food frequency questionnaire (FFQ) during prenatal visits [26]. This FFQ has previously been validated within the Korean population, with validation studies indicating that the mean energy and nutrient intakes estimated by the calibrated FFQ were comparable to those obtained from dietary records [29]. The version utilized in our study included a total of 138 items, an expansion from the original 113 items, to account for seasonal fruit consumption in Korea. The questionnaire comprised a list of foods with standard serving sizes and offered nine frequency categories: three times daily, twice daily, once daily, five or six times weekly, three or four times weekly, once or twice weekly, two or three times monthly, once monthly, and never or seldom. Portion sizes were classified into three categories: small, medium, and large, corresponding to designated units (e.g., cup or bowl). Daily intake was calculated by multiplying the frequency of consumption of each food or beverage item by its respective portion size. Nutrient and food intakes were quantified using a computer-aided nutritional analysis program (CAN-Pro version 4.0; Korean Nutrition Society, Seoul, Republic of Korea).

To evaluate the nutritional adequacy of the diet, the nutrient adequacy ratio (NAR) was calculated as a percentage for each of 15 nutrients: protein, vitamin A, vitamin B1, vitamin B2, niacin, vitamin B6, folate, vitamin B12, vitamin C, calcium, phosphorus, iron, zinc, iodine, and selenium. The NAR is computed using the following formula: NAR = participant’s daily intake of a nutrient/recommended nutrient intake (RNI) of that nutrient, according to sex and age. The NAR for a specific nutrient is the ratio of the participant’s intake to the RNI for that nutrient, with a maximum value of 1. An NAR of less than 1 signifies that nutrient consumption is below the RNI, while an NAR equal to 1 indicates that nutrient intake meets or exceeds the RNI [30]. To assess overall dietary adequacy, the mean adequacy ratio (MAR) was calculated as the average of the 15 NARs, using the formula: MAR = ΣNAR (each clipped at 1)/number of nutrients. In this study, nutrient intake was evaluated based on the 2020 Korean Dietary Reference Intakes.

### 2.3. KHEI

The KHEI is classified into three categories: adequacy, moderation, and energy balance, comprising a total of 14 components [25]. The adequacy category determines whether the recommended intake of food and nutrients is met, consisting of eight components: frequency of breakfast consumption; mixed grain intake; total fruit intake; fresh fruit intake; total vegetable intake; vegetable intake, excluding kimchi and pickles; meat, fish, egg, and bean intake; and milk and dairy product intake. Fruit intake is categorized into two items: total fruit intake, encompassing all fresh, canned, and dried fruits (excluding fruit juice), and fresh fruit intake, in accordance with the dietary guidelines. Similarly, vegetable intake includes two items: total vegetable intake, incorporating all vegetables, mushrooms, and seaweed, and vegetable intake excluding salted vegetables such as kimchi and pickles. The moderation category evaluates the consumption of restricted foods and nutrients, comprising three components: the ratio of energy intake from saturated fatty acids, sodium intake, and the ratio of energy intake from sugars and beverages. The final category—energy balance—assesses the distribution of energy and macronutrient intake, consisting of three components: the ratios of energy derived from carbohydrates and fat, as well as total energy intake. The overall KHEI score is 100 points, with each component assigned an equal weight of 10 points; sub-components of fruits and vegetables receive 5 points each. For breakfast consumption, based on the FFQ questionnaire, 10 points are awarded when the reported rice intake frequency is three times per day, while 0 points are assigned otherwise.

### 2.4. Statistical Analysis

Statistics are presented as frequencies and mean values ± standard deviations (SD) for maternal sociodemographic characteristics and lifestyle variables, stratified by KHEI score quartiles. The analysis examined sociodemographic characteristics, lifestyle variables, energy and nutrient intakes, NARs, MARs, and the distribution of KHEI components in relation to KHEI score quartiles. Continuous demographic and socioeconomic variables, divided into quartiles, were assessed for significant differences using one-way analysis of variance, while categorical variables were evaluated using the chi-square test. Differences in continuous variables between groups were assessed using Student’s *t*-test. To evaluate trends in energy and nutrient intakes, NARs, MARs, and KHEI component scores according to KHEI score quartiles, a general linear model was employed, without adjustment for confounding variables. We conducted multivariable logistic regression analyses to examine the association between maternal diet quality, assessed using the KHEI, and the risk of SGA birth. Covariates adjusted in the final model included maternal age, pre-pregnancy BMI, maternal education level, household income, total energy intake (log-transformed), and urinary cotinine levels (log-transformed). Women diagnosed with gestational hypertension or gestational diabetes were excluded at baseline. The analytical sample size varied slightly across models owing to missing dietary intake, covariate, or infant birth weight data.

Data on pregnancies conceived through assisted reproductive technologies (ART) and previous SGA deliveries were unavailable in the dataset and could not be included in the analysis. Notably, women with pregnancy complications, such as gestational hypertension and gestational diabetes, had already been excluded during cohort selection, thereby ensuring that these conditions did not confound the associations examined.

All statistical analyses were performed using Statistical Analysis System software (version 9.4; SAS v9.4, SAS Institute Inc., Cary, NC, USA). A two-sided *p*-value < 0.05 was considered statistically significant.

## 3. Results

### 3.1. General Characteristics of the Subjects

The demographic characteristics of the 1158 mothers and infants included in this analysis are summarized according to maternal KHEI score quartiles (Table 1). Higher KHEI scores were associated with increased maternal age, parity, education level, and household income, whereas urinary cotinine levels decreased. The distribution of infant sex varied across KHEI score quartiles. Although the proportion of mothers taking supplements tended to increase with higher dietary quality scores, this trend was not statistically significant (*p* = 0.058). The baseline characteristics of included versus excluded participants are presented in Supplementary Table S2.

### 3.2. Energy and Nutrient Intake by KHEI Score Quartile

Table 2 presents the energy and nutrient intakes of pregnant women according to KHEI score quartile. Energy intake was significantly higher in the highest KHEI score quartile (Q4) than in the lowest (Q1), with values of 2311.9 ± 605.0 and 1694.2 ± 881.7 kcal, respectively. Among macronutrients, energy derived from carbohydrates increased with higher KHEI scores, whereas that from protein and fat decreased. Notably, the percentage of energy intake from sugars did not exhibit significant variation across dietary quality scores. Daily nutrient intake (g/day), with the exception of fat, significantly increased with higher KHEI scores.

### 3.3. NAR and MAR in Pregnant Women

Table 3 delineates the NARs and MARs for individual nutrients in relation to the KHEI scores of pregnant women. An increase in the KHEI score correlated with a significant rise in the NAR for all nutrients. Specifically, the intake of vitamin B1, vitamin B12, phosphorus, zinc, and selenium met or exceeded the RNI in the highest quartile (Q4).

### 3.4. KHEI Component Scores According to KHEI Quartile

Table 4 displays the scores of KHEI components when stratified by KHEI score quartiles (Table 4). The mean dietary quality score for the 1158 participants was 65.2 (SD = 10.0). Thirteen out of the 14 dietary components constituting the KHEI score demonstrated significant differences as dietary quality improved. The only component that did not exhibit differences across the quartiles was “Percentage of energy from carbohydrates” (*p* for trend = 0.063). On comparing the three categories of Adequacy, Moderation, and Balance within the KHEI framework, all categories revealed significant score differences (*p* for trend < 0.001).

### 3.5. Associations Between Maternal KHEI Scores and SGA Infants

On comparing total KHEI scores between pregnant women with SGA infants and those without, the former displayed slightly higher scores, although this difference was not statistically significant (65.3 ± 9.9 vs. 63.0 ± 10.3). However, the score for “Total fruit intake” was significantly higher in the group of pregnant women without SGA infants (*p* = 0.035) (Supplement Table S1).

Table 5 illustrates the association between dietary quality indicators and SGA outcomes. The prevalence of SGA among pregnant women decreased as dietary quality increased from the lowest quartile (Q1) to the highest (Q4) (Q1: 8.3%, Q4: 4.1%). Specifically, the risk of having an SGA infant decreased by approximately 50% as dietary quality progressed from the first to the fourth quartile (OR = 0.478; 95% CI: 0.234–0.976; *p* for trend = 0.032). Even after adjusting for covariates, a significant association between dietary quality and SGA risk persisted (AOR = 0.448; 95% CI: 0.201–0.997; *p* for trend = 0.031).

## 4. Discussion

This study investigated the dietary intake of pregnant women in Korea and examined the association between maternal diet quality, assessed using the KHEI, and the risk of delivering an SGA infant. Our findings indicate that as the maternal dietary quality score increased, overall nutrient intake also improved. Furthermore, we identified a correlation between higher dietary quality scores and a reduced risk of giving birth to an SGA infant.

Previous research on the association between maternal dietary quality during pregnancy and fetal growth has corroborated our findings. For instance, the New Hampshire Birth Cohort Study in the United States (US) revealed an inverse linear trend, demonstrating that the probability of SGA births decreased as maternal dietary quality scores (Alternative Healthy Eating Index [AHEI]-2010) increased [31]. Likewise, Mexican pregnant women with elevated dietary quality scores (HEI-2015) exhibited a lower risk of SGA births and enhanced birth nutritional status markers. Additionally, a study involving pregnant women in Spain found that those with the highest AHEI scores had the lowest risk of delivering infants with fetal growth restriction [32]. Conversely, some studies have reported no significant associations between maternal dietary quality and fetal outcomes, such as SGA or fetal growth, suggesting potential heterogeneity arising from variations in population characteristics, dietary assessment tools, and confounding factors such as maternal pre-pregnancy weight and smoking, among others [33,34]. In our study, we appropriately adjusted for maternal age, education, income, and urinary cotinine levels in multivariate models, thereby enhancing the robustness of the observed association.

Various countries have developed diverse dietary assessment indices to evaluate dietary habits, focusing on disease prevention and the improvement of national dietary patterns [21,22,23,24]. The KHEI is a dietary quality assessment tool specifically designed to reflect the foods and nutrients consumed by Koreans. Research indicates that higher KHEI scores are significantly associated with disease prevention and dietary improvement [25,35]. Previous studies have demonstrated a positive correlation between the KHEI score and nutrient intake, particularly for fiber and vitamin C. A superior KHEI score reflects a balanced diet, and high dietary quality is linked to an increased consumption of assorted nutrients [36]. In our study, groups with higher dietary quality scores exhibited increased intakes of fiber and all micronutrients, aligning with previous research findings. Furthermore, the scores for components such as “Have breakfast,” “Mixed grain intake,” “Total fruit intake,” “Total vegetable intake,” “Meat, fish, egg, and bean intake,” and “Milk and dairy product intake” in the fourth KHEI quartile increased compared with those in the first quartile. These results support the association of enhanced nutrient intake with higher KHEI scores.

During pregnancy, total energy intake increases to support fetal growth and maternal homeostasis, accompanied by heightened nutrient requirements [37]. To evaluate the nutritional status of pregnant women, we utilized the NAR and MAR, which assess both individual nutrient adequacy and overall dietary quality. The NAR for all nutrients increased alongside KHEI scores, indicating that higher dietary quality correlates with significantly enhanced nutrient intake. In the highest KHEI quartile, most nutrients reached levels ≥0.8, suggesting adequate intake. However, vitamin A was notably deficient, at only 69% of the RNI, indicating inadequate consumption [38]. This is consistent with previous findings indicating low vitamin A intake among adults aged ≥19 years in Korea [39]. Adequate vitamin A intake during pregnancy is crucial for alleviating the risk of SGA, emphasizing the necessity for a balanced intake across all nutrients, rather than preferentially focusing on any single nutrient [40]. Korean diets predominantly comprise plant-based sources of provitamin A, such as green and orange vegetables, which are rich in beta-carotene. Nevertheless, the low bioavailability and conversion efficiency of these compounds may contribute to the low NAR for vitamin A, even among women with otherwise high-quality diets [39]. This highlights the importance of considering bioefficacy in nutrient surveillance during pregnancy.

In addition to nutrient adequacy, the physiological mechanisms by which dietary quality influences fetal growth should be explored. High-quality dietary patterns—characterized by an increased consumption of antioxidant-rich foods, fiber, and unsaturated fats, alongside a decreased intake of trans fats, saturated fats, and added sugars—have been associated with reduced oxidative stress and systemic inflammation in both pregnant and non-pregnant populations [11]. Considering that elevated oxidative stress during pregnancy is linked to restricted fetal growth and lower birth weight, antioxidant-rich diets may help mitigate these effects and reduce the risk of SGA. These mechanisms may partially explain why higher KHEI scores were associated with a lower SGA risk in our study.

Although the average KHEI score among pregnant women in this study (65.2 out of 100) is comparable to that of the general Korean female population (64.8) [25], caution is warranted in its interpretation, as the KHEI was originally designed for non-pregnant adults. By applying the KHEI in a vast Korean birth cohort, this study extends evidence from Western populations to an East Asian context. It demonstrates the value of a culturally adapted index that reflects Korean dietary characteristics, such as rice-based meals, vegetable side dishes, and seaweed consumption. Nevertheless, this limitation suggests that it may not adequately capture the specific nutritional requirements associated with pregnancy. Consequently, relying solely on this index to evaluate maternal dietary quality may overlook critical aspects of dietary adequacy that are particularly significant during this period. Notably, in the US, the AHEI for Pregnancy (AHEI-P) was specifically developed to address pregnancy-related dietary needs, including calcium, iron, and folate. The AHEI-P has a maximum score of 90 points, with previous studies reporting an average score of 61.4 [20]. This score surpasses the average KHEI score observed among Korean pregnant women in our study (65.2 out of 100 on the KHEI scale). In contrast, when pregnant women in the US were assessed using the AHEI-2010, a general dietary quality index comparable to the KHEI, the average score was 52.4 out of 100, which is lower than the average KHEI score of Korean pregnant women [31]. These contrasting patterns suggest that while Korean pregnant women may exhibit superior dietary quality when assessed using a general index, they may yield lower scores when assessed using a tool specifically designed for pregnancy. Taken together, these comparisons underscore the imperativeness of utilizing pregnancy-specific dietary quality indices to accurately assess maternal nutrition. General indices like the KHEI may inadequately reflect vital aspects of nutritional needs during pregnancy, potentially masking significant deficiencies. Therefore, developing a version of the KHEI tailored to the unique requirements of pregnant women would likely enhance the validity and sensitivity of dietary quality assessments during pregnancy in Korea.

From a public health perspective, our findings suggest that improving maternal diet quality should be prioritized within prenatal care programs. Incorporating KHEI-based assessments into routine prenatal nutrition counseling could assist in identifying women at risk of poor diet quality and adverse birth outcomes. Tailored nutrition counseling based on culturally relevant dietary patterns may enhance the effectiveness of prenatal interventions and contribute to improved birth outcomes.

To more effectively evaluate the dietary quality of pregnant women in Korea, (i) formulating a dietary quality score that incorporates important dietary factors specific to this population and (ii) conducting further research are imperative. In addition, ongoing efforts are required to promote and maintain optimal dietary quality among pregnant women.

This study has certain limitations. First, the dietary quality score calculation utilized a dichotomous scoring system for the component “Have breakfast,” assigning it a score of 0 or 10. This KHEI scoring method contrasts with the scoring of other components and might have hindered the accurate measurement of the overall KHEI score. Second, participants lacking complete information on dietary intake or infant birth weight, as well as those deemed unsuitable for analysis, were excluded in accordance with the study design. The exclusion of these participants might have influenced the estimated dietary intake and nutrient consumption reported in this study. We observed a difference in household income between included and excluded participants, which may introduce selection bias. Although household income was adjusted for in all multivariable analyses and the main findings were largely unchanged, residual bias cannot be fully excluded. Considering that the KHEI was originally developed for the general adult population, it may not adequately address pregnancy-specific nutritional needs, such as increased requirements for folate, iron, and calcium. Third, the lack of data on ART and SGA history might have limited our ability to account for these significant risk factors. Fourth, while women with gestational hypertension or gestational diabetes were excluded at baseline, other unmeasured complications could still have introduced residual confounding.

Furthermore, while the FFQ is a valuable tool for epidemiological studies—ranking individuals based on intake levels and identifying extreme consumption patterns—it has inherent limitations [41,42]. The FFQ requires participants to select food intake frequencies at discrete intervals, potentially leading to the underestimation or overestimation of food intake quantities [43].

Despite these limitations, this study is, to the best of our knowledge, the first to examine the relationship between maternal dietary quality assessed using the KHEI and SGA risk among pregnant women in Korea. It extends international evidence to an East Asian population, highlights the imperativeness of culturally adapted indices in maternal health research, and underlines the potential impact of improving maternal dietary quality on pregnancy outcomes. Furthermore, the study benefits from a relatively large sample size, providing robust insights.

## 5. Conclusions

Our findings indicate that higher maternal dietary quality, as assessed using the KHEI, is associated with improved nutrient intake and a reduced risk of SGA births among Korean pregnant women. These findings underscore the importance of culturally adapted dietary indices and emphasize the potential of enhancing maternal diet quality as a modifiable factor for improving perinatal outcomes. Further research is required to refine pregnancy-specific diet quality tools and validate their predictive utility across diverse populations.

## Figures and Tables

**Table 1:** Characteristics of the study participants according to quartiles of maternal diet quality in the Mothers and Children’s Environmental Health (MOCEH) study ^1^.

		Quartile of Korean Health Eating Index				
Characteristics	n	Q1 (n = 289)	Q2 (n = 290)	Q3 (n = 290)	Q4 (n = 289)	*p* Value ^2^
Pregnant women						
Age, y	1158	29.4 ± 3.7	30.2 ± 3.5	30.5 ± 3.6	30.8 ± 3.8	<0.001
Height, cm	1142	161.2 ± 4.9	161.4 ± 4.8	161.1 ± 4.8	161.1 ± 4.4	0.906
Prepregnancy weight, kg	1141	54.5 ± 8.0	55.3 ± 8.1	55.2 ± 8.8	55.0 ± 7.5	0.655
Prepregnancy BMI (kg/m^2^)	1135	20.9 ± 2.7	21.2 ±3.0	21.2 ± 3.1	21.2 ± 2.8	0.522
Parity, n (%)	995					
0		137 (47.4)	140 (48.3)	104 (35.9)	106 (36.7)	<0.001
≥1		101 (35.0)	114 (39.3)	144 (49.7)	149 (51.6)	
No response		51 (17.7)	36 (12.4)	42 (14.5)	34 (11.8)	
Education level, n (%)	1139					
≤high school		109 (37.7)	70 (24.1)	72 (24.8)	57 (19.7)	<0.001
<University		56 (19.4)	69 (23.8)	48 (16.6)	47 (16.3)	
≥University		118 (40.8)	147 (50.7)	165 (56.9)	181 (62.6)	
No response		6 (2.1)	4 (1.4)	5 (1.7)	4 (1.4)	
Household income (USD), n (%)	1126					
≤2000		101 (35.0)	76 (26.2)	62 (21.4)	71 (24.6)	0.007
2000–4000		146 (50.5)	147 (50.7)	169 (58.3)	147 (50.9)	
≥4000		34 (11.8)	59 (20.3)	51 (17.6)	63 (21.8)	
No response		8 (2.8)	8 (2.8)	8 (2.8)	8 (2.8)	
Nutritional supplements, n (%)	1029					
No		120 (41.5)	123 (42.4)	113 (39.0)	111 (38.4)	0.058
Yes		124 (42.9)	140 (48.3)	144 (49.7)	154 (53.3)	
No response		45 (15.6)	27 (9.3)	33 (11.4)	24 (8.3)	
Urinary cotinine levels, μg/g creatinine	1073	43.3 ± 263.6	15.3 ± 94.2	7.9 ± 44.0	9.4 ± 80.4	0.018
Infant						
Sex, n (%)	1158					
Boys		151 (52.3)	137 (47.2)	178 (61.4)	139 (48.1)	0.002
Girls		138 (47.8)	153 (52.8)	112 (38.6)	150 (51.9)	
Gestational age, d	1158	274.9 ± 10.8	274.0 ± 10.8	274.8 ± 9.0	274.7 ± 10.4	0.677
Birth length, cm	627	50.4 ± 2.8	50.3 ± 2.6	50.1 ± 2.6	50.6 ± 2.3	0.441
Birth weight, g	1154	3233.3 ± 456.4	3251.0 ± 417.3	3285.8 ± 420.1	3280.0 ± 402.2	0.401

^1^ Values are means ± SDs or n (%). SD, standard deviations; Q, quartile; BMI, body mass index. ^2^ Chi-square test was used for categorical variables, and one-way analysis of variance (ANOVA) was used for continuous variables.

**Table 2:** Energy and nutrient intakes according to quartiles of maternal diet quality in the Mothers and Children’s Environmental Health (MOCEH) study ^1^.

		Quartile of Korean Health Eating Index				
Energy and Nutrient	Total (n = 1158)	Q1 (n = 289)	Q2 (n = 290)	Q3 (n = 290)	Q4 (n = 289)	*p* for Trend ^2^
KHEI range		0–58.67	58.67–65.28	65.28–72.69	72.69–85.79	
Energy (kcal)	2040.6 ± 764.2	1694.2 ± 881.7	2045.9 ± 783.1	2110.2 ± 621.8	2311.9 ± 605.0	<0.001
% Total energy/day:						
Carbohydrates	65.1 ± 6.4	62.7 ± 8.8	64.2 ± 6.1	66.0 ± 5.0	67.4 ± 3.7	<0.001
Total sugar	13.3 ± 5.1	12.9 ± 5.9	14.3 ± 5.8	13.2 ± 4.8	13.0 ± 3.7	0.546
Protein	14.0 ± 2.4	14.4 ± 3.2	14.3 ± 2.5	13.7 ± 2.0	13.5 ± 1.6	<0.001
Total fat	21.4 ± 5.8	23.8 ± 8.0	22.1 ± 5.3	20.4 ± 4.4	19.0 ± 3.3	<0.001
Saturated fat	7.2 ± 2.2	8.2 ± 3.0	7.5 ± 2.0	6.8 ± 1.5	6.3 ± 1.2	<0.001
MUFA	6.8 ± 2.1	7.9 ± 2.9	7.1 ± 1.9	6.5 ± 1.6	6.0 ± 1.2	<0.001
PUFA	4.6 ± 1.4	4.8 ± 1.7	4.8 ± 1.3	4.5 ± 1.2	4.3 ± 1.0	<0.001
*N*-3 fatty acids	0.6 ± 0.2	0.6 ± 0.3	0.7 ± 0.3	0.6 ± 0.2	0.6 ± 0.2	0.213
*N*-6 fatty acids	4.0 ± 1.2	4.3 ± 1.5	4.1 ± 1.1	3.9 ± 1.1	3.7 ± 0.9	<0.001
*N*-6/*N*-3 ratio	6.4 ± 1.3	6.9 ± 1.6	6.4 ± 1.2	6.3 ± 1.1	6.1 ± 1.1	<0.001
Carbohydrates (g)	329.9 ± 119.1	258.9 ± 119.7	325.0 ± 116.8	346.6 ± 97.3	389.0 ± 103.0	<0.001
Total sugar (g)	68.4 ± 37.9	55.5 ± 39.8	72.9 ± 40.3	70.4 ± 39.1	74.9 ± 28.0	<0.001
Total dietary fiber (g)	19.0 ± 9.7	14.9 ± 10.7	19.9 ± 11.0	19.6 ± 7.9	21.5 ± 7.7	<0.001
Protein (g)	71.8 ± 32.0	62.4 ± 41.6	73.7 ± 32.6	72.8 ± 26.5	78.2 ± 21.9	<0.001
Total fat (g)	49.2 ± 25.6	47.7 ± 36.1	51.4 ± 25.0	48.7 ± 20.8	49.1 ± 16.2	0.826
Saturated fat (g)	16.5 ± 8.8	16.4 ± 12.6	17.2 ± 8.4	16.1 ± 7.2	16.1 ± 5.3	0.366
MUFA (g)	15.8 ± 8.6	15.8 ± 12.4	16.4 ± 8.1	15.5 ± 7.0	15.5 ± 5.4	0.380
PUFA (g)	10.6 ± 5.6	9.5 ± 7.3	11.1 ± 5.9	10.6 ± 4.5	11.1 ± 4.1	0.002
*N*-3 fatty acids (g)	1.5 ± 0.9	1.3 ± 1.0	1.6 ± 1.0	1.5 ± 0.7	1.6 ± 0.6	<0.001
*N*-6 fatty acids (g)	9.2 ± 4.9	8.4 ± 6.5	9.7 ± 5.1	9.3 ± 3.9	9.6 ± 3.6	0.012
Calcium (mg)	608.9 ± 297.6	504.3 ± 352.4	643.1 ± 318.6	612.4 ± 261.7	675.7 ± 210.9	<0.001
Phosphorus (mg)	1081.1 ± 446.8	903.1 ± 545.5	1109.7 ± 466.9	1101.8 ± 369.7	1209.6 ± 314.2	<0.001
Iron (mg)	15.1 ± 6.7	12.5 ± 7.9	15.6 ± 7.1	15.5 ± 5.4	16.8 ± 5.3	<0.001
Sodium (mg)	3228.6 ± 1711.1	2819.1 ± 2072.6	3462.1 ± 1968.2	3250.3 ± 1355.1	3382.2 ± 1220.6	<0.001
Potassium (mg)	2938.3 ± 1313.4	2336.3 ± 1464.0	3032.7 ± 1399.3	3026.2 ± 1081.1	3357.5 ± 1045.0	<0.001
Vitamin A (μg RAE)	428.6 ± 263.5	345.3 ± 301.0	454.5 ± 290.0	432.4 ± 225.8	482.2 ± 205.7	<0.001
Vitamin B1 (mg)	2.0 ± 0.8	1.7 ± 1.0	2.0 ± 0.8	2.0 ± 0.7	2.2 ± 0.6	<0.001
Vitamin B2 (mg)	1.4 ± 0.7	1.2 ± 0.8	1.5 ± 0.7	1.4 ± 0.5	1.5 ± 0.4	<0.001
Niacin (mg NE)	13.8 ± 6.2	11.6 ± 7.7	14.2 ± 6.4	14.1 ± 5.1	15.3 ± 4.6	<0.001
Vitamin C (mg)	105.2 ± 70.3	70.8 ± 51.9	110.2 ± 74.6	110.6 ± 69.6	129.1 ± 69.8	<0.001
Vitamin D (μg)	2.3 ± 1.7	1.9 ± 1.8	2.4 ± 2.0	2.3 ± 1.6	2.5 ± 1.5	<0.001
Vitamin E (mg α-TE)	12.9 ± 6.7	11.3 ± 8.5	13.6 ± 7.3	13.0 ± 5.3	13.8 ± 4.6	<0.001
Vitamin K (μg)	168.7 ± 113.5	132.2 ± 129.4	174.4 ± 130.3	176.4 ± 94.9	191.9 ± 83.8	<0.001
Vitamin B6 (mg)	1.4 ± 0.6	1.2 ± 0.7	1.5 ± 0.6	1.5 ± 0.5	1.6 ± 0.5	<0.001
Folate (μg DFE)	520.1 ± 242.7	403.2 ± 251.0	536.9 ± 261.7	539.5 ± 211.7	600.7 ± 197.5	<0.001
Vitamin B12 (μg)	7.8 ± 4.6	6.3 ± 4.8	8.3 ± 5.4	7.9 ± 4.0	8.5 ± 3.6	<0.001
Pantothenate (mg)	2.6 ± 1.4	2.2 ± 1.6	2.8 ± 1.4	2.7 ± 1.2	2.9 ± 1.1	<0.001
Biotin (μg)	13.1 ± 7.2	10.2 ± 7.2	13.7 ± 8.2	13.4 ± 6.2	14.9 ± 6.3	<0.001
Magnesium (mg)	71.0 ± 43.6	56.7 ± 48.5	74.2 ± 43.5	73.8 ± 38.8	79.2 ± 39.8	<0.001
Zinc (mg)	10.9 ± 4.2	9.0 ± 5.2	10.9 ± 4.1	11.1 ± 3.5	12.4 ± 3.1	<0.001
Copper (μg)	0.9 ± 0.5	0.8 ± 0.6	1.0 ± 0.5	0.9 ± 0.4	1.0 ± 0.5	<0.001
Manganese (mg)	3.9 ± 1.6	3.0 ± 1.6	3.9 ± 1.5	4.1 ± 1.2	4.7 ± 1.5	<0.001
Iodine (μg)	292.5 ± 308.5	235.3 ± 373.3	300.9 ± 301.8	319.6 ± 308.6	314.2 ± 226.8	0.002
Selenium (μg)	98.1 ± 42.0	81.9 ± 48.8	99.6 ± 46.5	100.7 ± 33.0	110.3 ± 31.7	<0.001

^1^ Values are means ± SD. SD, standard deviations; KHEI, Korean Healthy Eating Index; MUFA, Monounsaturated fatty acid; PUFA, Polyunsaturated fatty acids. ^2^ *p*-for-trend was assessed by modeling the mean value of the quartile in the general linear model (GLM).

**Table 3:** The NAR and MAR ratio according to the quartiles of diet quality in the Mothers and Children’s Environmental Health (MOCEH) study ^1^.

	Quartile of Korean Health Eating Index				
	Q1 (n = 289)	Q2 (n = 290)	Q3 (n = 290)	Q4 (n = 289)	*p* for Trend ^2^
NAR(%)					
Protein	0.83 ± 0.19	0.96 ± 0.09	0.99 ± 0.04	0.99 ± 0.03	<0.001
Vitamin A	0.48 ± 0.29	0.62 ± 0.26	0.62 ± 0.24	0.69 ± 0.21	<0.001
Vitamin B1	0.94 ± 0.12	0.99 ± 0.03	1.00 ± 0.01	1.00 ± 0.00	<0.001
Vitamin B2	0.75 ± 0.25	0.89 ± 0.16	0.91 ± 0.13	0.96 ± 0.07	<0.001
Niacin	0.68 ± 0.24	0.83 ± 0.18	0.87 ± 0.14	0.93 ± 0.11	<0.001
Vitamin C	0.60 ± 0.30	0.78 ± 0.25	0.83 ± 0.22	0.88 ± 0.18	<0.001
Vitamin B6	0.70 ± 0.23	0.86 ± 0.16	0.90 ± 0.13	0.96 ± 0.09	<0.001
Folic acid	0.78 ± 0.22	0.93 ± 0.13	0.96 ± 0.09	0.98 ± 0.06	<0.001
Vitamin B12	0.97 ± 0.10	1.00 ± 0.02	1.00 ± 0.03	1.00 ± 0.00	<0.001
Calcium	0.61 ± 0.28	0.77 ± 0.22	0.78 ± 0.20	0.86 ± 0.15	<0.001
Phosphorus	0.87 ± 0.17	0.98 ± 0.05	1.00 ± 0.02	1.00 ± 0.01	<0.001
Iron	0.71 ± 0.22	0.87 ± 0.15	0.91 ± 0.12	0.96 ± 0.09	<0.001
Zinc	0.84 ± 0.18	0.96 ± 0.08	0.99 ± 0.03	1.00 ± 0.03	<0.001
Iodine	0.74 ± 0.31	0.89 ± 0.20	0.91 ± 0.17	0.95 ± 0.12	<0.001
Selenium	0.90 ± 0.15	0.98 ± 0.07	1.00 ± 0.01	1.00 ± 0.01	<0.001
MAR (%)	0.76 ± 0.18	0.89 ± 0.11	0.91 ± 0.08	0.94 ± 0.06	<0.001

^1^ Values are means ± SD. SD: standard deviations; NAR, Nutrient adequacy ratio; MAR, Mean adequacy ratio. ^2^ *p*-for-trend was assessed by modeling the mean value of the quartile in the general linear model (GLM).

**Table 4:** Korean Health Eating Index component scores by quartiles of maternal diet quality in the Mothers and Children’s Environmental Health (MOCEH) study ^1^.

	Quartile of Korean Health Eating Index				
Component	Q1 (n = 289)	Q2 (n = 290)	Q3 (n = 290)	Q4 (n = 289)	*p* for Trend ^2^
Total KHEI score	52.0 ± 5.7	62.5 ± 1.9	68.9 ± 2.1	77.5 ± 3.2	<0.001
Adequacy	21.0 ± 7.9	27.6 ± 5.7	31.7 ± 4.8	39.5 ± 4.4	<0.001
Have breakfast	0.5 ± 2.2	1.2 ± 3.3	3.2 ± 4.7	8.5 ± 3.6	<0.001
Mixed grains intake	0.3 ± 0.4	0.5 ± 0.5	0.5 ± 0.5	0.6 ± 0.6	<0.001
Total fruits intake	1.8 ± 1.4	2.7 ± 1.6	3.2 ± 1.4	3.7 ± 1.4	<0.001
Fresh fruits intake	3.0 ± 1.6	3.9 ± 1.4	4.4 ± 1.1	4.6 ± 0.9	<0.001
Total vegetables intake	2.9 ± 1.4	3.6 ± 1.3	3.8 ± 1.2	4.2 ± 1.0	<0.001
Vegetables intake excluding Kimchi and pickled vegetables intake	2.7 ± 1.4	3.5 ± 1.3	3.7 ± 1.3	4.1 ± 1.0	<0.001
Meat, fish, eggs and beans intake	3.2 ± 1.4	3.7 ± 1.2	3.8 ± 1.1	4.0 ± 0.9	<0.001
Milk and milk products intake	6.4 ± 3.5	8.2 ± 2.8	8.5 ± 2.5	9.3 ± 1.7	<0.001
Moderation	22.1 ± 6.8	23.0 ± 5.5	25.3 ± 4.3	26.2 ± 2.8	<0.001
Percentage of energy from saturated fatty acid	5.0 ± 4.4	6.7 ± 3.6	8.4 ± 2.5	9.4 ± 1.4	<0.001
Sodium intake	7.8 ± 3.1	6.9 ± 3.2	7.2 ± 2.6	6.9 ± 2.4	0.002
Percentage of energy from sweets and beverages	9.2 ± 2.1	9.4 ± 1.7	9.7 ± 1.0	9.9 ± 0.5	<0.001
Balance	9.0 ± 3.9	12.1 ± 2.8	12.5 ± 2.5	12.2 ± 2.3	<0.001
Percentage of energy from carbohydrate	3.2 ± 1.9	3.9 ± 1.6	3.8 ± 1.5	3.5 ± 1.4	0.063
Percentage of energy from fat	3.8 ± 1.8	4.7 ± 1.0	4.7 ± 0.9	4.9 ± 0.6	<0.001
Energy intake	2.0 ± 2.2	3.5 ± 2.1	4.0 ± 1.9	3.8 ± 1.9	<0.001

^1^ Values are means ± SD. SD: standard deviations; KHEI, Korean Healthy Eating Index. ^2^ *p*-for-trend was assessed by modeling the mean value of the quartile in the general linear model (GLM).

**Table 5:** OR and AOR for SGA according to the quartiles of Korean Healthy Eating Index in the Mothers and Children’s Environmental Health (MOCEH) study ^1^.

	Quartile of Korean Healthy Eating Index				
	Q1	Q2	Q3	Q4	*p* for Trend
KHEI range	27.190–58.570	58.571–65.295	65.296–72.682	72.683–85.793	
SGA n/total n	(24/289)	(18/290)	(15/290)	(12/289)	
OR (95% CI)	1 (ref.)	0.731 (0.388–1.378)	0.602 (0.309–1.173)	0.478 (0.234–0.976)	0.032
AOR (95% CI) ^2^	1 (ref.)	0.809 (0.413–1.587)	0.570 (0.270–1.205)	0.448 (0.201–0.997)	0.031

^1^ AOR, adjusted odds ratio; Q, quartile; SGA, small-for-gestational-age. ^2^ Adjusted for maternal age, prepregnancy BMI, maternal education level, family income, maternal urinary cotinine concentration (log-transformed), and maternal energy intake (log-transformed). Adjustment data were available for 1051 pregnant women.

## Data Availability

Data described in the manuscript, code book, and analytic code will not be made available because approval of these activities was not obtained in the informed consent form.

## Supplementary Materials

The following supporting information can be downloaded at: https://www.mdpi.com/article/10.3390/nu17193056/s1; Supplemental Table S1. Differences in maternal KHEI components according to SGA infant pregnancies. Supplemental Table S2. Baseline characteristics of participants included versus excluded from the analysis.

## Abbreviations

The following abbreviations are used in this manuscript:

ART	Assisted Reproductive Technologies
BMI	Body mass index
FFQ	Food frequency questionnaire
HPLC	High-performance liquid chromatography
KDRIs	Korean Dietary Reference Intakes
KHEI	Korean Healthy Eating Index
MAR	Mean adequacy ratio
MOCEH	Mothers and Children’s Environmental Health study
MUFA	Monounsaturated fatty acid
NAR	Nutrient adequacy ratio
PUFA	Polyunsaturated fatty acids
RNI	Recommended Nutrient Intake
SGA	Small-for-gestational-age
