# Supplementary material for: Maternal Diet Quality Assessed Using the Korean Healthy Eating Index and Risk of Small-for-Gestational-Age Infants: Findings from the Mothers and Children’s Environmental Health (MOCEH) Study
Source: Nutrients. 2025 Sep 25;17(19):3056. doi: 10.3390/nu17193056 (PMC12526471; doi:10.3390/nu17193056)
Supplement: Supplementary file 1 [file nutrients-17-03056-s001.zip › nutrients-3860372-supplementary.pdf]

Supplemental Table S1 Differences in maternal KHEI components according to SGA infant pregnancies[1]

Component	SGA (-) (n=1089)	SGA (+) (n=69)	*P*-value[2]
Total KHEI score	65.3 ± 9.9	63.0 ± 10.3	0.063
**Adequacy**	30.1 ± 8.9	28.0 ± 8.8	0.063
Have breakfast	3.4 ± 4.7	2.8 ± 4.5	0.279
Mixed grains intake	0.5 ± 0.5	0.5 ± 0.5	0.809
Total fruits intake	2.9 ± 1.6	2.5 ± 1.6	**0.035**
Fresh fruits intake	4.0 ± 1.4	3.7 ± 1.6	0.078
Total vegetables intake	3.6 ± 1.3	3.5 ± 1.4	0.613
Vegetables intake excluding Kimchi and pickled vegetables intake	3.5 ± 1.4	3.5 ± 1.4	0.809
Meat, fish, eggs and beans intake	3.7 ± 1.2	3.7 ± 1.3	0.777
Milk and milk products intake	8.1 ± 2.9	7.8 ± 3.1	0.316
**Moderation**	24.2 ± 5.3	23.6 ± 6.7	0.504
Percentage of energy from saturated fatty acid	7.4 ± 3.6	7.1 ± 3.7	0.481
Sodium intake	7.2 ± 2.8	6.9 ± 3.5	0.477
Percentage of energy from sweets and beverages	9.6 ± 1.5	9.6 ± 1.2	0.668
**Balance**	11.5 ± 3.2	11.5 ± 3.5	0.863
Percentage of energy from carbohydrate	3.6 ± 1.7	3.9 ± 1.5	0.110
Percentage of energy from fat	4.5 ± 1.2	4.6 ± 1.3	0.727
Energy intake	3.4 ± 2.2	3.1 ± 2.3	0.249

[1] Values are means ± SDs

[2] Student's t-test for continuous variables were used to compare the difference between the groups

Supplemental Table S2 Baseline characteristics of participants included versus excluded from the analysis

Variable	n	Included (n=1,158)	Excluded (n=593)	*P*-value[2]
Age, y		30.5 ±3.7	30.3±3.9	0.579
Height, cm		161.2 ±4.7	161.3±4.9	0.650
Prepregnancy weight, kg		54.8±7.5	55.1±7.6	0.762
Prepregnancy BMI (kg/m^2)		21.1±2.7	21.2±2.9	0.844
Parity, n(%)				0.042
0		388(49.6)	166(54.8)	
≥1		395(50.5)	137(45.2)	
Education level, n(%)				
≥University		591(75.5)	234(77.2)	0.122
Household income (USD), n(%)				0.014
≤2000		80(10.2)	72(23.8)	
2000-4000		523(66.8)	151(49.8)	
≥4000		180(23.0)	80(26.4)	

[1] Values are means ± SDs or n (%). SD, standard deviations; BMI, body mass index

[2] Chi-square test was used for categorical variables, and Student's t-test for continuous variables were used for continuous variables
